# Divergent Profile of Emerging Cutaneous Leishmaniasis in Subtropical Brazil: New Endemic Areas in the Southern Frontier

**DOI:** 10.1371/journal.pone.0056177

**Published:** 2013-02-14

**Authors:** Mariel Asbury Marlow, Marise da Silva Mattos, Maria Ernestina Makowiecky, Iriane Eger, Andre Luiz Rossetto, Edmundo Carlos Grisard, Mário Steindel

**Affiliations:** 1 Department of Microbiology, Immunology and Parasitology, Universidade Federal de Santa Catarina, Florianópolis, Brazil; 2 Instituto de Biofísica Carlos Chagas Filho, Universidade Federal de Rio de Janeiro, Rio de Janeiro, Brazil; 3 Department of Zoonoses, Diretoria de Vigilância Epidemiológica, Secretaria Estadual de Saúde de Santa Catarina, Florianópolis, Brazil; 4 Center for Health Sciences, Universidade do Vale do Itajaí, Itajaí, Brazil; Technion-Israel Institute of Technology Haifa, Israel

## Abstract

**Background:**

Although known to be highly endemic in the Amazon regions of Brazil, the presence of cutaneous leishmaniasis (CL) in the subtropical southern part of the country has largely been ignored. This study was conducted to demonstrate CL is emerging in the Brazilian state of Santa Catarina, as well as to characterize the epidemiological profile and *Leishmania* species involved.

**Methodology/Principal Findings:**

For this cross-sectional study, data from all CL cases from Santa Catarina, Brazil, reported to the Brazilian National Notifiable Diseases Information System from 2001 to 2009 were investigated. Amplification of the kDNA minicircle conserved region followed by restriction fragment length polymorphism (PCR-RFLP) was conducted to screen for *Leishmania* species present in patient biopsy. Overall, 542 CL cases were reported, with majority resulting from autochthonous transmission (n = 401, 73.99%) and occurring in urban zones (n = 422, 77.86%). Age, gender, zone of residence, origin of case, clinical form and case outcome were found to differ significantly by region. Imported cases were over seven times more likely to relapse (95% CI 2.56–21.09). Mapping of cases revealed new endemic areas in northeastern Santa Catarina with two species present. With the exception of three *L. (Leishmania) amazonensis* cases (1.20%), majority of PCR positive samples were found to be *L. (Viannia) braziliensis* (n = 248, 98.80%).

**Conclusions/Significance:**

CL is now endemic in the state of Santa Catarina, Brazil, with case profiles varying significantly by region. *L. (V.) braziliensis* has been identified as the predominant species in the region.

## Introduction

Being the fifth largest country in the world, the territory of Brazil spans vastly different geographical biomes, including the tropical Amazon regions of the north, the plains in the central region and the subtropical south, where in some cities temperatures have been known to reach below 0°C in the winter months. Overall, a higher incidence of most infectious diseases can be found in the northern and northeastern regions where geographical, economic, and social factors aid in their transmission [1]. However, despite the contrasting population and geographical characteristics between the southern region with the country as a whole, migration of human, animal, and possibly vector populations has imported several of these classically “tropical” diseases from other regions to the subtropical south of Brazil [2]–[6].

Already known to emerge in various regions around the world and highly adaptable to new vectors and hosts [7], [8], leishmaniasis is one of these infectious diseases which has been introduced into South Brazil over the past few decades [9]–[11]. Leishmaniasis is a vector-borne neglected tropical disease spread by female sand flies and caused by protozoan parasites belonging to genus *Leishmania* [12]. Brazil is among one of seven countries reporting 90% of cutaneous leishmaniasis (CL) cases worldwide [12] and reports an average of 28,000 CL cases per year [13]. In the 1980s, only nineteen states in Brazil had reported CL cases, yet by 2003, all twenty–seven Brazilian states presented autochthonous CL transmission [13], demonstrating the continued geographic expansion of the disease across the country. *Leishmania* parasites are present in a variety of ecological niches and can infect a large range of hosts and vectors [14]. In Brazil, several sylvatic and synanthropic mammals have been incriminated as reservoirs as well as the domestic dog, which possibly contributes to the noted increase in the urbanization of the disease transmission cycle [15]–[17]. Ten *Leishmania* species from two different subgenera have been found to cause CL in humans in the New World, with *L. (Viannia) braziliensis* followed by *L. (Leishmania) amazonensis* and *L. (V.) guyanensis* being the most widely distributed of these species in Brazil [14]. Additionally, leishmaniasis presents several other clinical forms beyond CL, including mucocutaneous and visceral forms, all of which have specific relationships with one to many different *Leishmania* species [18]. Thus, the complexity of the disease complicates control efforts to combat its expansion and results in a lack of proven effective control methods for leishmaniasis today [7]. Furthermore, when leishmaniasis emerges into a new area, characterization of epidemiological, vectorial and clinical parameters can be difficult and parasite characteristics in a specific region cannot be assumed to be similar to *Leishmania* spp. found in neighboring regions. The extent of this complexity was demonstrated in a study conducted by Falqueto et al. (2003), in which even different genotypes of *L. (V.) braziliensis* were found to have specific transmission cycles within the state of Espírito Santo, Brazil [19].

Roughly similar in land size to Portugal and with a population of over 6 million [20], Santa Catarina has been experiencing a increase in population in the past few decades, largely due to migration of workers from different regions of Brazil hoping to benefit from the state’s industrial, agricultural and information technology sectors [21], [22]. Since the 1980s, this southern Brazilian state regularly has received imported CL cases. However, in 1990, São Thiago and Guida published the first report of autochthonous CL cases in the western region of Santa Catarina [23]. In their study, out of fourteen confirmed cases, eleven were considered autochthonous. However, despite continued reporting of autochthonous CL cases to the Brazilian National Notifiable Diseases Information System [24], no investigation has been conducted to determine if CL has emerged and/or is endemic in this region, nor have the current epidemiological profile, species prevalence and species and case geographical distributions been determined. Without the establishment of epidemiologic parameters and species characterization, this neglected tropical disease will continue to remain without intervention, leaving the state, as well as neighboring Argentina and other southern Brazilian states, vulnerable to further emergence and outbreaks. Moreover, species characterization is the first piece of the puzzle to establishing a transmission cycle in the region as well as an important factor for diagnosis and treatment. Therefore, this study was conducted to demonstrate CL is an emerging neglected tropical disease in the state of Santa Catarina, Brazil, to characterize the epidemiological profile and species involved and to discuss the implications of this emergence for future research and control strategies.

## Methods

### Study Area and Data Collection

Data used for this retrospective cross-sectional study were restricted to cases reported in the state of Santa Catarina, Brazil. The political territory of Santa Catarina covers a total land area of 95,703.49 km^2^ and shares a western border with Argentina. During the study period, the state maintained an average population of 5,763,329 residents [20]. Being situated in the subtropical climate zone, temperatures in the state range from an average low of 8.5°C (47.3°F) in the winter, with even snow occurring in some cities, to an average high of 28.7°C (83.7°F) in the summer [25]. The costal eastern region is split from the western region by the Serra do Mar mountain range, creating six mesoregions with varying geographical characteristics. For this study, the mesoregions were grouped into two regions based on similarities in geography, biome, watershed and population migration. The northeastern (NE) regions of Grande Florianópolis, Norte Catarinense and Vale do Itajaí are located to the east of the Serra do Mar mountain range, are on or with proximity to the Atlantic coast, mainly consist of Atlantic Forest vegetation, are supplied by the South Atlantic water basin, and experience internal and external migration mainly due to tourism and urbanization. The region also includes numerous international tourist destinations. The southern and western (SW) regions of Oeste Catarinense, Serrana and Sul Catarinense contain or lie to the west of the Serra Geral and/or Serra do Mar mountain ranges, consist of both Tropical Forest and Araucaria Forest, are supplied by the Uruguay River basin, and experience mainly external migration from other Brazilian states for the purpose of rural labor.

Case data were collected as part of the National Epidemiological Surveillance System of Brazil (Sistema de Informação de Agravos de Notificação- SINAN). All suspected cutaneous leishmaniasis cases by clinical profile were confirmed by parasitological methods (Giemsa stained smears and/or PCR). Only cases found positive by at least one parasitological method were considered as confirmed cases and reported to SINAN for follow-up. Case reporting began with collection of patient information in an interview by a trained public health official during evaluation at health centers. Next, data from confirmed cases were entered into the SINAN database by the Epidemiological Surveillance System municipality unit. During the interview, patients were questioned regarding any previous travel outside of the state and/or country in the two years preceding disease presentation in order to determine if the case was imported or resulted from autochthonous transmission within the state. Once electronically transcribed, data were sent to the Division of Epidemiologic Surveillance of the Santa Catarina State Health Department (Diretoria de Vigilância Epidemiológica, Secretaria Estadual de Saúde de Santa Catarina- DIVE), where the reports were reviewed for consistency and completeness by state officials who specialize in vector-borne diseases and any questionable or missing information was followed-up on a case by case basis. Through evaluation of patient history, cases were considered relapse cases if they had been previously diagnosed and treated for CL before or within the study period, were classified as cured based on remission of disease symptoms and/or negative PCR and then presented again with disease symptoms and positive smears, cultures, and/or PCR. For this study, data from all cases of CL in Santa Catarina, Brazil reported to SINAN from January 1^st^, 2001, to December 31^st^, 2009, were investigated. Proper confidentiality procedures dictated by DIVE were observed to protect the confidentiality of patient information. The present study was approved by the Universidade Federal de Santa Catarina (UFSC) Ethics Committee (003/07/CPSH).

### DNA Extraction

DNA for *Leishmania* species characterization was extracted from patient biopsy samples collected for diagnosis before initiation of treatment. Biopsy samples were placed in 500 µl of lysis buffer and incubated with proteinase K (20 mg/ml) overnight at 42°C. The resulting lysates were then subjected to phenol–chloroform extraction as described elsewhere [26]. Following isopropanol precipitation, the pellet was washed twice with 500 µl of cold 70% ethanol, resuspended in 50 µl of ultra-pure water containing RNAse A (10 mg/ml), incubated at 37°C for 1 h and then stored at −20°C until use. To monitor for possible contamination during DNA extraction, a negative extraction control was used.

### *Leishmania* Species Molecular Characterization

Amplification of the kDNA minicircle conserved region followed by restriction fragment length polymorphism (PCR-RFLP) was used to determine *Leishmania* species present in patient biopsy [27]. The 120 bp DNA band corresponding to a fragment of *Leishmania* minicircle k-DNA was PCR-amplified using primers 150 [5′- GGG (G/T)AG GGG CGT TCT (G/C)CG AA -3′] and 152 [5′- (G/C)(G/C)(G/C) (A/T)CT AT(A/T) TTA CAC CAA CCC C -3′]. DNA from confirmed *Leishmania* negative patients and DNA-free water were used as negative controls and DNA from reference strains *L.* (*V*.) *braziliensis* (MHOM/BR/75/M-2904) and *L.* (*L.*) *amazonensis* (IFLA/BR/67/PH8) were used as positive controls. Following amplification and isopropanol precipitation, 5 µL of PCR products were separately digested at 37°C with *Hae*III and *Ava*I (New England Biolabs) restriction enzymes overnight according to manufacturer’s instructions. Restriction fragments were separated by electrophoresis in 10% SDS-PAGE gels. Samples considered positive for *L.* (*V.*) *braziliensis* were those which produced 40 bp and 80 bp fragments when digested with *Hae*III and were not digested by *Ava*I. Samples considered positive for *L.* (*L*.) *amazonensis* were those which produced 38 bp and 78 bp fragments when digested with *Ava*I and were not digested by *Hae*III.

Additionally, when properly prepared biopsy or aspirates were available for *Leishmania* parasite isolation, samples were seeded in Schneider medium supplemented with 5% of heat inactivated fetal bovine serum and incubated at 26°C. Positive cultures were cryopreserved in liquid nitrogen. All isolated strains were deposited in the *Leishmania* Collection of the Oswaldo Cruz Institute (Coleção de *Leishmania* do Instituto Oswaldo Cruz - CLIOC, WDCM731, http://clioc.ioc.fiocruz.br) international cryobank in Rio de Janeiro, Brazil, and characterized by multilocus enzyme electrophoresis (MLEE) following standard protocols set forth by CLIOC.

### Statistical Analysis and Mapping

Descriptive statistics were produced for all cases. Chi-squared tests, or Fisher’s exact tests when appropriate, were used to determine significant associations between northeastern and southwestern regions for case demographic and clinical characteristics. Bivariate logistic regression analysis predicting probability of case relapse was performed for each potential risk factor, including being male, living in a rural zone, 50 years or age or older, mucocutaneous, and imported from other Brazilian states. Factors found to be significant in the bivariate analysis then were included in the multivariate logistic regression analysis. The Hosmer and Lemeshow goodness of fit test was used to assess whether there was evidence for lack of fit in the logistic regression model. Data were analyzed using R version 2.13.2 (R Development Core Team, Vienna, Austria). Unadjusted odds ratios, adjusted odds ratios, 95% confidence intervals, and p-values are reported. All cases were geocoded by address and maps were produced using ArcGIS 10 (ESRI, Redlands, CA, USA).

## Results

**Figure 1:**
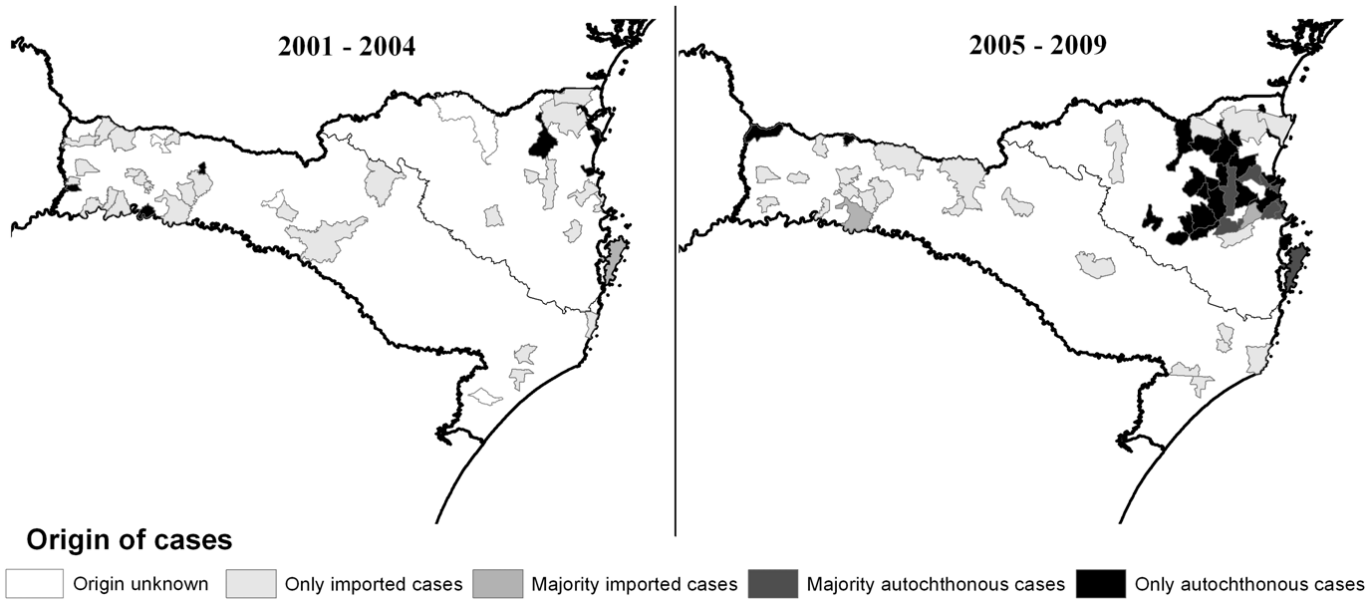
Comparison of maps demonstrating cutaneous leishmaniasis case origin by municipality between the time periods of 2001–2004 and 2005–2009.

Of the 669 suspected cases of cutaneous leishmaniasis in the state of Santa Catarina, Brazil, during the study period, 542 (81.02%) cases were confirmed positive by at least one parasitological test and reported to the National Epidemiological Surveillance System of Brazil for epidemiological follow-up. Incidence of CL cases was found to rise from 14 cases in 2001 to a peak of 166 cases in 2006, and then gradually decreased to 48 cases in 2009. Overall, majority of cases were white (n = 365, 90.96%), male (n = 365, 67.34%), and lived in urban zones (n = 422, 77.86%) (Table 1). The average patient age was 37 years, with 34 (6.27%) cases occurring in children 10 years of age or younger (Table 1). While 73.99% (n = 401) of all cases were autochthonous in origin, mapping of municipalities by case origin and separating by the time periods of 2001 to 2004 and 2005 to 2009 demonstrated a geographical change in case origin overtime (Fig. 1). From 2001 until 2004, cases were geographically dispersed and mostly imported from other states. However, from 2005 until 2009, a drastic increase in autochthonous cases in the northeastern (NE) region can be observed, with cases in the southwestern (SW) region remaining mainly imported. When compared by chi-squared test, case origin was found to differ significantly between the regions (p = <0.0001), with majority of cases in the NE region being of autochthonous transmission (n = 395, 84.22%) and majority of cases in the SW region being imported (n = 55, 75.34%) (Table 1).

**Table 1:** Comparison of case demographics and clinical characteristics of cutaneous leishmaniasis cases between the northeastern and southern/western regions of Santa Catarina from 2001 to 2009 by chi-squared test or Fisher’s exact test.

Characteristic		NortheasternRegion no. (%)	SouthwesternRegion no. (%)	p-value	Total no. (%)
Age group	0–10	33 (7.04)	1 (1.37)	0.0208	34 (6.27)
	11–20	69 (14.71)	8 (10.96)		77 (14.21)
	21–30	75 (15.99)	14 (19.18)		89 (16.42)
	31–40	94 (20.04)	13 (17.81)		107 (19.74)
	41–50	77 (16.42)	11 (15.07)		88 (16.24)
	50–65	96 (20.47)	14 (19.18)		110 (20.30)
	>65	24 (5.12)	11 (15.07)		35 (6.46)
	Unknown	1 (0.21)	1 (1.37)		2 (0.37)
Gender	Female	170 (36.25)	7 (9.59)	<0.0001	177 (32.66)
	Male	299 (63.75)	66 (90.41)		365 (67.34)
Race or ethnicity	Indigenous	0 (0.00)	0 (0.00)	0.2653	0 (0.00)
	White	426(90.83)	67 (91.78)		493 (90.96)
	Black	10 (2.13)	3 (4.11)		13 (2.40)
	Asian	1 (0.21)	0 (0.00)		1 (0.18)
	Mixed race	21 (4.48)	0 (0.00)		21 (3.87)
	Unknown	11 (2.35)	3 (4.11)		14 (2.58)
Zone of residence	Urban	372 (79.32)	50 (68.49)	0.0003	422 (77.86)
	Rural	53 (11.30)	21 (28.77)		74 (13.65)
	Periurban	31 (6.61)	1 (1.37)		32 (5.90)
	Unknown	13 (2.77)	1 (1.37)		14 (2.58)
Origin of case	Autochthonous	395 (84.22)	6 (8.22)	<0.0001	401 (73.99)
	Imported	47 (10.02)	55 (75.34)		102 (18.82)
	Unknown	27 (5.76)	12 (16.44)		39 (7.20)
Clinical form	Cutaneous	455 (97.01)	61 (83.56)	<0.0001	516 (95.20)
	Mucocutaneous	14 (2.99)	12 (16.44)		26 (4.80)
Case outcome	Cured case	446 (95.10)	64 (87.67)	0.0294	510 (94.10)
	Relapse case	18 (3.84)	7 (9.59)		25 (4.61)
	Unknown	5 (1.07)	2 (2.74)		7 (1.29)
Total		469 (86.53)	73 (13.47)		542 (100.00)

**Figure 2:**
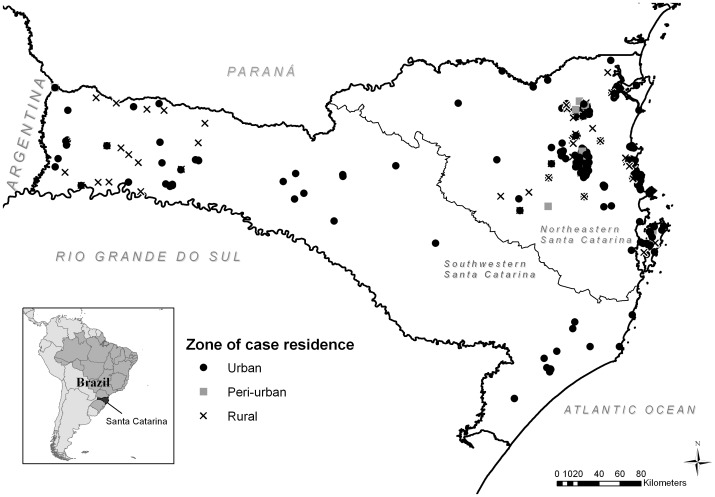
Map of cutaneous leishmaniasis cases by zone of residence (urban/peri-urban/rural), Santa Catarina, Brazil (n = 452, 2001–2009).

Furthermore, age group, gender, zone of residence, clinical form, and case outcome also were found to have a significant association with region of diagnosis (p = 0.0208, <0.0001, 0.0003, <0.0001, and 0.0294, respectively) (Table 1). In the NE region, the ages of cases were more evenly distributed across the age groups than in the SW region where cases tended to be of working adult age. Nearly all cases in children 10 years of age or younger occurred in the NE region (n = 33/34, 97.06%). The NE region presented a nearly 1 to 2 ratio of females to males, with a significantly higher percentage of women (n = 177, 32.66%) than in the SW region (n = 7, 9.59%) (p = <0.0001). Majority of cases in both regions were urban; however, NE regions contained a higher percentage of periurban cases (n = 31, 6.61%) and SW region contained a higher percentage of rural cases (n = 21, 28.77%). When mapped by zone of case residence, both urban and rural cases were heavily clustered in the NE region, while cases were more dispersed in the SW region (Fig. 2).

**Figure 3:**
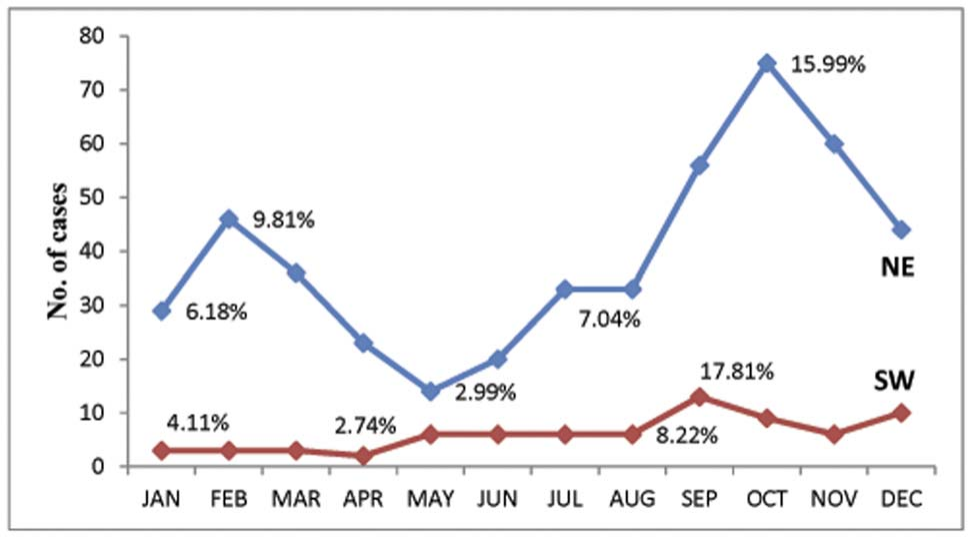
Seasonal distribution of cutaneous leishmaniasis in Santa Catarina, Brazil, by region; NE = Northeastern region, SW = Southern and western regions (n = 452, 2001–2009).

Seasonality of CL cases was found to differ between regions as well (Fig. 3). The SW peak season occurred in August through September with 8.22% and 17.81% of cases occurring during this time, respectively. However, two peak seasons in February and October (9.81% and 15.99% of cases, respectively) were observed in the NE region, with October being the more dominate of the two peaks.

For the clinical characteristic of cases, the SW region presented a significantly higher percentage of mucocutaneous cases (n = 12, 16.44%), a more severe clinical form of the disease, compared to the NE region. A significantly higher percentage of relapse cases was observed in the SW region as well. Investigation into risk factors for CL relapse in the state revealed being 50 years of age or older, originating from transmission outside of the state (imported) or having mucocutaneous clinical form of the disease were significant risk factors. In the multivariate logistic regression, imported cases were found to be almost seven times significantly more likely to be a relapse case even after adjusting for the other risk factors of patient age and clinical form of CL (95% CI 2.56–21.09) (Table 2). The logistic model was considered acceptable based on failure to reject the null hypothesis in the Hosmer and Lemeshow goodness of fit test (χ^2^ = 0.035, p = 0.9828). Majority of these imported cases originated from the Amazonian state of Mato Grosso (n = 54, 51.43%).

**Table 2:** Multivariate logistic regression predicting cutaneous leishmaniasis case relapse in Santa Catarina, Brazil.

Potential risk factor	Unadjusted odds ratio	95% CI	Adjusted odds ratio	95% CI
Male	1.95	0.72–5.28	−	−
Living in a rural zone	1.16	0.39–3.48	−	−
50 years or age or older	2.44*	1.09–5.49	1.52	0.55–4.19
Mucocutaneous	10.05*	3.75–26.94	1.82	0.43–7.78
Imported from other Brazilian states	8.96*	3.27–24.51	7.35†	2.56–21.09

* p<0.05,

† p<0.001.

**Figure 4:**
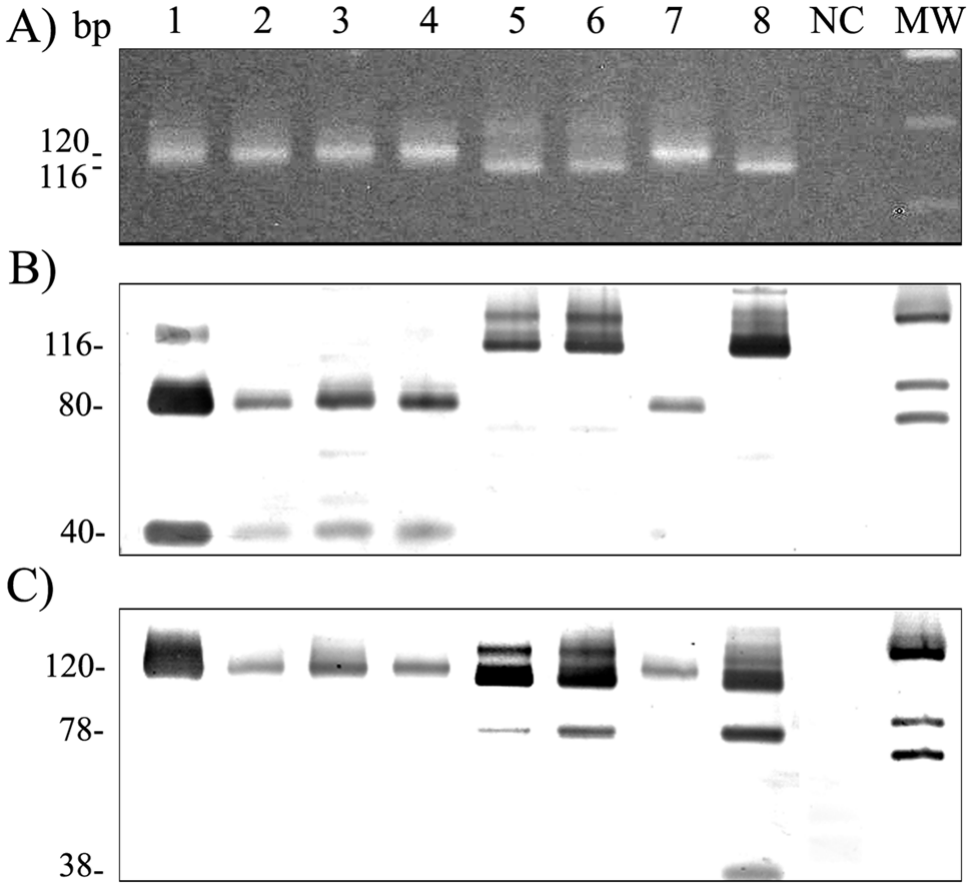
Molecular characterization of *Leishmania* species present in biopsy sample. (A) PCR amplification products of the *Leishmania* kDNA minicircle conserved region from cutaneous leishmaniasis cases in Santa Catarina, Brazil, stained with ethidium bromide; Restriction fragment length polymorphism (RFLP) of the amplified the kDNA minicircle conserved region digested with restriction enzymes *Hae*III (B) and *Ava*I (C), and analyzed by electrophoresis in 10% polyacrylamide gels with silver staining; Lane 1–2 = *L. (V.) braziliensis* cases from the northeastern region; Lane 3–4 = *L. (V.) braziliensis* cases from the southern and western regions, Lane 5–6 = *L. (L.) amazonensis* cases, Lane 7 = reference strain *L. (V.) braziliensis* (MHOM/BR/75/M-2904), Lane 8 = reference strain *L. (L.) amazonensis* (IFLA/BR/67/PH8), NC = negative control, MW = molecular weight.

Among the 542 CL cases confirmed by parasitological tests, diagnosis by PCR was performed for 331 (61.1%) patients. The remaining cases were confirmed by Giemsa stained smears. Positive PCR biopsy sample was available for PCR-RFLP analysis for 251 (46.31%) patients (Fig. 4). Majority of these samples were found to be positive for *L.* (*V*.) *braziliensis* (n = 248, 98.80%). Three cases, one imported in 2001 from the state of Mato Grosso and two autochthonous cases in 2002 and 2005, were found to be positive for *L.* (*L*.) *amazonensis*. All cases RFLP positive for *L.* (*L*.) *amazonensis* were located in the NE region. For these same 251 patients, parasite isolation by culture was possible for 39 patient samples. All 39 strains have been deposited in the *Leishmania* Collection of the Oswaldo Cruz Institute (CLIOC). MLEE characterization performed by CLIOC was consistent with the kDNA PCR-RFLP results found in this study, with one *L. (L.) amazonensis* strain and 38 *L. (V.) braziliensis* strains being deposited.

## Discussion

Evidence presented in this study has confirmed the emergence of cutaneous leishmaniasis in Santa Catarina, South Brazil. However, case profiles between the two main regions of the state were found to differ significantly, demonstrating this emergence is not uniform. While cases in the southwestern region continue to be dispersed and mainly imported, the northeastern region is now endemic for CL in over three main urban zones. The high relapse rate in imported cases as well as the high rate of mucosal cases in the SW region are important public health concerns.

The mucocutaneous form of leishmaniasis represents a more virulent form of the disease, which can lead to serious disfiguration and is more difficult to treat [12], [28]. Although other species have been incriminated as casual agents of mucosal leishmaniasis, *L.* (*V.*) *braziliensis*, the main species of *Leishmania* found in this study, is the most commonly associated species with this clinical form in Brazil [12], [29]–[31]. While the percentage of mucosal cases in the state (5.25%) is similar to the percentage of mucosal cases overall in Brazil (6.61% in 2009) [24], the percentage of mucosal cases in the SW region of Santa Catarina (18.67%) alone is nearly three times higher than this national average. Given the significant association between case origin and clinical form, this higher percentage of mucosal cases reflects the higher percentage of imported cases in the SW region. Majority of these imported cases originated from the state of Mato Grosso, where *L.* (*V*.) *braziliensis* has been confirmed as the most prevalent species of *Leishmania* [32] and an average of 6.2% of annual CL cases are mucosal [24].

Species found in this study conform with those found in a previous study where *L.* (*L*.) *amazonensis* was identified in two case isolates from 1987 in the western region and *L.* (*V*.) *braziliensis* was identified in one isolates from 1996 from the western region and one isolate from 1997 in the northeastern region of Santa Catarina [33]. However, this study has now confirmed *L.* (*V*.) *braziliensis,* the most widely distributed species in Brazil [14], is also the predominant species in the region and responsible for the emergence of the disease in Santa Catarina. Interestingly, *L. (L.) amazonensis* was also identified in this study from two autochthonous cases, despite no record of its known vector species (*Lutzomyia (Nyssomyia) flaviscutellata, Lu. (N.) olmeca olmeca* and *Lu. (N.) reducta*) having been reported in the state to date [29]. Only *Lu. (N.) neivai* has been found to be naturally infected with *L.* (*V*.) *braziliensis* in Santa Catarina [34]. Other sandfly species previously observed in Santa Catarina were *Lu. (Pintomyia) fischeri* and *Lu. (Psychodopygus) ayrozai* [35]. Multiple climate change prediction models conducted in 2003 have demonstrated the likelihood of dramatic improvements in habitat condition in southern Brazil for *Lu. whitmani*, as well as, increasing suitability in habitat for *Lu. intermedia* and *Lu. migonei* [5]. All three phlebotomine species are proven vectors of *L.* (*V*.) *braziliensis* [29].

Cases of CL in Santa Catarina in the past decade have consisted of both urban and rural cases. However, in the now endemic northeastern region, nearly 80% of cases are urban, following a rising trend in urbanization of the once rural disease in states across Brazil [24], [36]–[38]. This study is unable to conclude vector competence in domestic habitats of southern Brazil, and thus, it cannot be confirmed if these urban cases are contracting the disease in domestic settings or returning from rural areas of transmission. However, the relatively higher proportion of women and children under 10 years of age of these CL cases in the NE region is suggestive of a peri-domestic or domestic transmission cycle. In the state of Paraná, the state just north of Santa Catarina, a majority of urban cases (59.2% of all cases reported from 1987 to 2004) has been reported as well [39]. However, with 78.08% urban and 5.80% peri-urban cases, this study has revealed that Santa Catarina now represents the state with one of the highest proportions of urban cases in Brazil [24]. Additionally, mapping of cases demonstrated CL clusters around urban centers in the endemic areas.

One of the most distinct differences between the two regions of Santa Catarina was the rate of autochthonous versus imported cases. In the NE region, the majority of cases resulted from autochthonous transmission, supporting the claim of endemicity in the region. In the SW regions, the majority of imported cases reflected the expected migration of labor workers to this region. Additionally, most of these cases originated in Mato Grosso, where the majority of labor exchange with Santa Catarina occurs. This high rate of imported cases from Mato Grosso was also reported in 1997, when 52.1% of the 23 imported cases reported between 1994 and 1997 were found to be imported from Mato Grosso as well [10]. In addition, the presence of only one case in an individual with less than 10 years of age in the western region supports the hypothesis that these cases were mainly migrant workers from other states. Of concern is the higher likelihood that these cases will be mucosal and/or relapse cases, as was demonstrated in the multivariate logistic regression analysis of this study. These imported cases were over seven times significantly more likely to be a relapse case, even after accounting for clinical form and being 50 years of age or older. Thus, these cases require improved case monitoring and further prospective studies are needed to determine the cause of this higher relapse rate.

Seasonality of cases differed between the NE and SW regions. The two CL case peak seasons of February and October coincided with the two peak tourism seasons in the NE region of the state. This overlap is a major public health concern since a higher rate of cases combined an influx of population increases the risk of transmission and could result in geographical dispersion of leishmaniasis, specifically transmission of the predominant parasite species in the region *L.* (*V*.) *braziliensis*.

Overall, given the different disease patterns between the NE and the SW regions, control strategies and treatment approaches should be modified by region to adequately prevent further emergence and future CL outbreaks in Santa Catarina.

An important limitation of this study was the potential underreporting of CL. Given leishmaniasis is not recognized as an endemic disease in the state, many physicians are unaware of its presence and the disease is not commonly included as a differential diagnosis. Some cases may have been treated as bacterial or fungal infections and/or self-cured and were not reported to SINAN. However, this only further supports the main objective of this study to prove the emergence of the disease and suggests the problem of leishmaniasis may be even greater than the current evidence demonstrates. Future studies should seek to assess and/or include unreported cases in their study designs as the characteristics of these cases may differ from those of reported cases.

Santa Catarina has not been widely recognized as a state endemic for leishmaniasis, which inhibits support for research and control efforts. Considered secondary to diseases with higher mortality and morbidity, CL case reporting may be delayed for months or even years, preventing the possibility of early interventions for outbreaks. Additionally, Santa Catarina has immense potential for tourism and is experiencing an increase in both domestic and international tourism each year [40]. The combination of new endemic areas with a high level of tourism could result in both national and global CL transmission from Santa Catarina.

Thus, as CL continues to expand in the state of Santa Catarina, research efforts into the causes of disease transmission and outbreaks, including determination of vector species and natural reservoirs, are needed in order to develop interventions and control strategies. Additionally, since CL requires a lengthy and costly treatment regimen [41], outbreaks could result in a serious burden on the public health care system. In conclusion, without increased pressure for more complete and efficient case reporting, funding for research, and development of interventions, the state is likely to endure further outbreaks and continued geographical expansion of this neglected tropical disease.
